# Burden of pneumococcal pneumonia requiring ICU admission in France: 1-year prognosis, resources use, and costs

**DOI:** 10.1186/s13054-020-03442-z

**Published:** 2021-01-10

**Authors:** Claire Dupuis, Ayman Sabra, Juliette Patrier, Gwendoline Chaize, Amine Saighi, Céline Féger, Alexandre Vainchtock, Jacques Gaillat, Jean-François Timsit

**Affiliations:** 1 AP-HP, Medical and Infectious Diseases Intensive Care Unit (MI2), Bichat-Claude Bernard University Hospital, 46 rue Henri Huchard, 75018 Paris, France; 2 Université de Paris, INSERM IAME, U1137, Team DesCID, 75018 Paris, France; 3 Medical ICU, Gabriel-Montpied University Hospital, Clermont-Ferrand, France; 4 Pfizer France, Paris, France; 5 HEVA, Lyon, France; 6 EMIBiotech, Paris, France; 7 Infectious Diseases Department, Annecy-Genevois Hospital, Annecy, France

**Keywords:** Community-acquired pneumonia, Pneumococcal pneumonia, *Streptococcus pneumoniae*, Intensive care unit, Long-term outcome, Direct costs, Comorbidities

## Abstract

**Background:**

Community-acquired pneumonia (CAP), especially pneumococcal CAP (P-CAP), is associated with a heavy burden of illness as evidenced by high rates of intensive care unit (ICU) admission, mortality, and costs. Although well-defined acutely, determinants influencing long-term burden are less known. This study assessed determinants of 28-day and 1-year mortality and costs among P-CAP patients admitted in ICUs.

**Methods:**

Data regarding all hospital and ICU stays in France in 2014 were extracted from the French healthcare administrative database. All patients admitted in the ICU with a pneumonia diagnosis were included, except those hospitalized for pneumonia within the previous 3 months. The pneumococcal etiology and comorbidities were captured. All hospital stays were included in the cost analysis. Comorbidities and other factors effect on the 28-day and 1-year mortality were assessed using a Cox regression model. Factors associated with increased costs were identified using log-linear regression models.

**Results:**

Among 182,858 patients hospitalized for CAP in France for 1 year, 10,587 (5.8%) had a P-CAP, among whom 1665 (15.7%) required ICU admission. The in-hospital mortality reached 22.8% at day 28 and 32.3% at 1 year. The mortality risk increased with age > 54 years, malignancies (hazard ratio (HR) 1.54, 95% CI [1.23–1.94], *p* = 0.0002), liver diseases (HR 2.08, 95% CI [1.61–2.69], *p* < 0.0001), and the illness severity at ICU admission. Compared with non-ICU-admitted patients, ICU survivors remained at higher risk of 1-year mortality. Within the following year, 38.2% (516/1350) of the 28-day survivors required at least another hospital stay, mostly for respiratory diseases. The mean cost of the initial stay was €19,008 for all patients and €11,637 for subsequent hospital stays within 1 year. One-year costs were influenced by age (lower in patients > 75 years old, p = 0.008), chronic cardiac (+ 11% [0.02–0.19], *p* = 0.019), and respiratory diseases (+ 11% [0.03–0.18], p = 0.006).

**Conclusions:**

P-CAP in ICU-admitted patients was associated with a heavy burden of mortality and costs at one year. Older age was associated with both early and 1-year increased mortality. Malignant and chronic liver diseases were associated with increased mortality, whereas chronic cardiac failure and chronic respiratory disease with increased costs.

**Trial registration:**

N/A (study on existing database)

## Background

Community-acquired pneumonia (CAP) causes a heavy burden of illness with high morbidity, mortality, and health-related costs [1–5]. In European countries with reliable coding systems, respiratory diseases are responsible for 15% of in-hospital deaths, with pneumonia being the second most important cause, and for 7% of hospital admissions, with pneumonia being the leading cause (2%). A recent review across Europe reported a still high incidence of CAP, of 68–7000 per 100,000 populations [4].

Among hospitalized CAP, the proportion of those requiring intensive care unit (ICU) admission ranges from 5 to 40% [6]; it was 22.7% in a recent US study [7]. Some scoring systems have been specifically set up to assist clinicians to identify patients who will require ICU admission [8, 9]. However, these scores are focused to identify patients with short-term mortality. The factors associated with an increased risk of long-term mortality, and the magnitude of the associated increase are poorly known.

In addition, the burden of in-hospital management of CAP, due to pneumococci (P-CAP) and other etiologies, as well as management of associated comorbidities, generates high costs. Although the short-term mortality and in-hospital costs of P-CAP in France have already been estimated [10], little is known about the associated 1-year survival, burden of hospital care, and costs. However, the evaluation of the overall burden associated with CAP, and more specifically P-CAP, is instrumental to guide public health strategies for CAP prevention, such as targeting pneumococcal or influenza vaccination to high-risk groups. This is of notable importance in France as a French study conducted in 2007–2012 showed only 2% of the patients admitted in the ICU for severe P-CAP were vaccinated against pneumococcal infections [11].

The objectives of this study were to ascertain the overall burden associated with ICU-admitted P-CAP in France in terms of in-hospital mortality and the associated direct costs, during both the initial hospital stay and within the year following the P-CAP onset.

## Methods

### Study objectives

The study objective aimed to evaluate the burden of severe P-CAP in France related to the initial hospital stay and subsequent hospital stays within the year following the pneumonia onset, regarding in-hospital mortality, and hospital-related costs. The study is based on an exhaustive administrative registry that collects data of all hospital stays in France.

### Database

Study data were obtained from the *Programme de Médicalisation des Systèmes d'Information* (PMSI) database, the health administrative database which records all discharges from public and private hospitals in France. This registry allows the chaining of all hospital stays of individual patients.

### Study population

All patients admitted to ICUs with a diagnosis of P-CAP were included. The International Statistical Classification of Diseases, 10th Revision (ICD-10), used for CAP diagnosis and comorbidities are detailed in the Online Data Supplement. A CAP-related hospitalization was any hospitalization with a principal diagnosis of pneumonia or a secondary diagnosis of pneumonia if the principal diagnosis was respiratory failure or sepsis. For P-CAP, the diagnoses were based on ICD-10 code J13: “Pneumonia due to *Streptococcus pneumoniae*” or B953 “*Streptococcus pneumoniae* as the cause of diseases classified elsewhere” and pneumonia. Patients with a hospital stay with a diagnosis of P-CAP within the previous 3 months were excluded. The severity of acute illness at ICU admission was reported through the Simplified Acute Physiology Score II (SAPS II), mandatory for all ICU stays. Additional file 1 provides additional details, including details on the variables related to the initial hospital stay and the subsequent stays within the following year (see Additional file 1: e-Tables 1 & 2).

**Table 1:** Characteristics of patients with pneumococcal community-acquired pneumonia admitted in intensive care unit

Characteristics of the patients	All (*n* = 1665)	28-day survivors (*n* = 1286)	One-year survivors (*n* = 1127)
Male gender	1108 (67%)	834 (65%)	730 (64.8%)
Age (median [Q1;Q3], years)	65 [55;76]	64 [53;75]	64 [53;74]
0–54 years old^a^	394 (23.7%)	345 (26.8%)	322 (28.6%)
55–64 years old	409 (24.6%)	327 (25.4%)	290 (25.7%)
65–75 years old	424 (25.5%)	316 (24.6%)	277 (24.6%)
≥ 76 years old	438 (26.3%)	298 (23.2%)	238 (21.1%)
Alcohol	429 (25.8%)	332 (25.8%)	289 (25.6%)
Tobacco	590 (35.4%)	476 (37.0%)	415 (36.8%)
No comorbidities	86 (5.1%)	82 (6.4%)	79 (7.0%)
At least one	1579 (94.8%)	1204 (93.6%)	1048 (93.0%)
Asplenia/hyposplenia	12 (0.7%)	9 (0.7%)	6 (0.5%)
Cancers / hemopathy	497 (29.8%)	347 (27.0%)	278 (24.7%)
Auto-immune disorders	146 (8.8%)	111 (8.6%)	97 (8.6%)
Organ transplant recipient^b^	267 (16.0%)	194 (15.1%)	157 (13.9%)
HIV	32 (1.9%)	21 (1.6%)	19 (1.7%)
Diabetes mellitus	415 (24.9%)	321 (25.0%)	275 (24.4%)
Chronic cardiac failure	518 (31.1%)	379 (29.5%)	297 (26.4%)
Chronic respiratory diseases^c^	861 (51.2%)	688 (53.5%)	594 (52.7%)
Renal diseases^d^	239 (14.4%)	172 (13.4%)	140 (12.4%)
Chronic renal insufficiency	202 (12.1%)	146 (11.4%)	118 (10.5%)
Liver diseases^e^	272 (16.3%)	170 (13.2%)	142 (12.6%)
Cirrhosis	165 (9.9%)	106 (8.2%)	88 (7.8%)
Liver failure	118 (7.1%)	61 (4.7%)	49 (4.3%)
SAPSII	48 (± 19)	45 (± 17)	44 (± 17)
Mechanical ventilation	1103 (66.2%)	789 (61.4%)	706 (62.6%)
Noninvasive ventilation	987 (59.3%)	818 (63.6%)	708 (62.8%)
Vasopressor use	1032 (62.0%)	728 (56.6%)	640 (56.8%)
Renal replacement therapy	227 (13.6%)	105 (8.2%)	94 (8.3%)
Length of initial stay in hospital (days)	23 (± 22)	24 (± 21)	24 (± 21)
Length of stay in ICU (days)	13 (± 16)	12 (± 15)	12 (± 15)

ICU: intensive care unit; HIV: human immunodeficiency virus; SAPS: Simplified Acute Physiological Score. Quantitative data: mean (± SD); qualitative data: number (%)

^a^Only two patients were less than 18: aged 9 and 17

^b^Organ transplant comprised solid organ transplant and hematopoietic stem cells transplant

^c^Chronic respiratory diseases included COPD, asthma, and respiratory insufficiency

^d^Chronic renal diseases included nephrotic syndrome, renal tubular and interstitial nephritis, renal tubular and interstitial lesions related to drugs or heavy metals, renal tubular and interstitial lesions related to other diseases, other renal tubular and interstitial lesions, chronic kidney disease, and renal failure

^e^The chronic liver diseases included cirrhosis (alcohol-related cirrhosis or from other causes, including liver fibrosis), liver insufficiency, liver inflammatory diseases, and the other chronic liver diseases

**Table 2:** Independent risk factors for an early versus late death of ICU-admitted patients with pneumococcal community-acquired pneumonia (note: other comorbidities did not independently impact the survival) (multivariate analysis, Cox model)

Independent risk factors (multivariate model)	Overall in-hospital mortality *N* = 1665				28-day in-hospital mortality *N* = 1665				One-year in-hospital mortality *N* = 1350			
	HR	95% CI		*p* Value	HR	95% CI		*p* Value	HR	95% CI		*p* Value
Age (years)				< 0.0001				< 0.0001				< 0.0001
0–54	1.00	–	–		1.00	–	–		1.00	–	–	
55–64	1.61	1.20-	2.16	0.0015	1.40	0.96-	2.05	0.0807	1.92	1.20-	3.06	0.0067
65–75	1.94	1.46-	2.57	< 0.0001	1.61	1.12-	2.32	0.0103	2.46	1.56-	3.87	0.0001
76 +	3.28	2.49-	4.32	< 0.0001	2.93	2.06-	4.16	< 0.0001	3.74	2.40-	5.84	< 0.0001
Female gender	1.08	0.90-	1.31	0.3998	1.01	0.79-	1.29	0.9586	1.21	0.91-	1.63	0.1945
Cancer / malignant hemopathy	1.60	1.34	1.91	< 0.0001	1.54	1.23-	1.94	0.0002	1.66	1.27-	2.18	0.0002
Chronic liver diseases	1.99	1.62	2.44	< 0.0001	2.53	1.96-	3.26	< 0.0001	1.80	1.28-	2.53	0.0008

### Costs evaluation

All hospital stays, encompassing those related to the initial ICU admission as well as subsequent hospitalizations within the following year (detailed in the supplement), were included in the cost analysis, with the details of inclusion stay costs (including the supplement related to ICU) and 1-year follow-up costs. Healthcare consumption items taken into account are described in Additional file 1.

Costings were restricted to direct in-hospital costs and determined from the perspective of the French social security system, according to the methodological rules of the French Technical Agency for Information on Hospitalization. Valuation of hospital stays included the 2014–2015 tariffs of the GHS (using upper and lower limits), with supplements and additional expenses related to drugs and medical devices. For private hospitals, clinicians' fees were added. Costs were expressed in 2016 Euros.

### Statistical methodology

All hospital stays were considered to be independent and included into the analyses. Standard descriptive statistics were performed for the whole cohort. Data were summarized as frequencies and percentages for categorical variables and medians with interquartile ranges or means with standard deviations for continuous variables.

Log-linear regression models were constructed to explore predictive factors associated with costs in adults. Factors significantly associated with the outcomes assessed in the univariate analysis (*p* value < 0.1) were included in the multivariate analysis with backward selection.

Cox regression was used to assess the effect of comorbidities on adult death. This method provides an estimate of the hazard ratio. The mortality was evaluated separately for early mortality (≤ 28 days after ICU admission, initial stay or the following) and late mortality (within the first year excluding the first 28 days) to respect proportional hazard hypotheses. Proportionality assumption of Cox models was checked graphically, and Schoenfeld residuals were calculated if clinically relevant.

The *p* values < 0.05 were considered significant. Data were analyzed using R (versions 3.2.3 and 3.6.1; R Core Team, Vienna, Austria).

## Results

### Study population

**Fig. 1:**
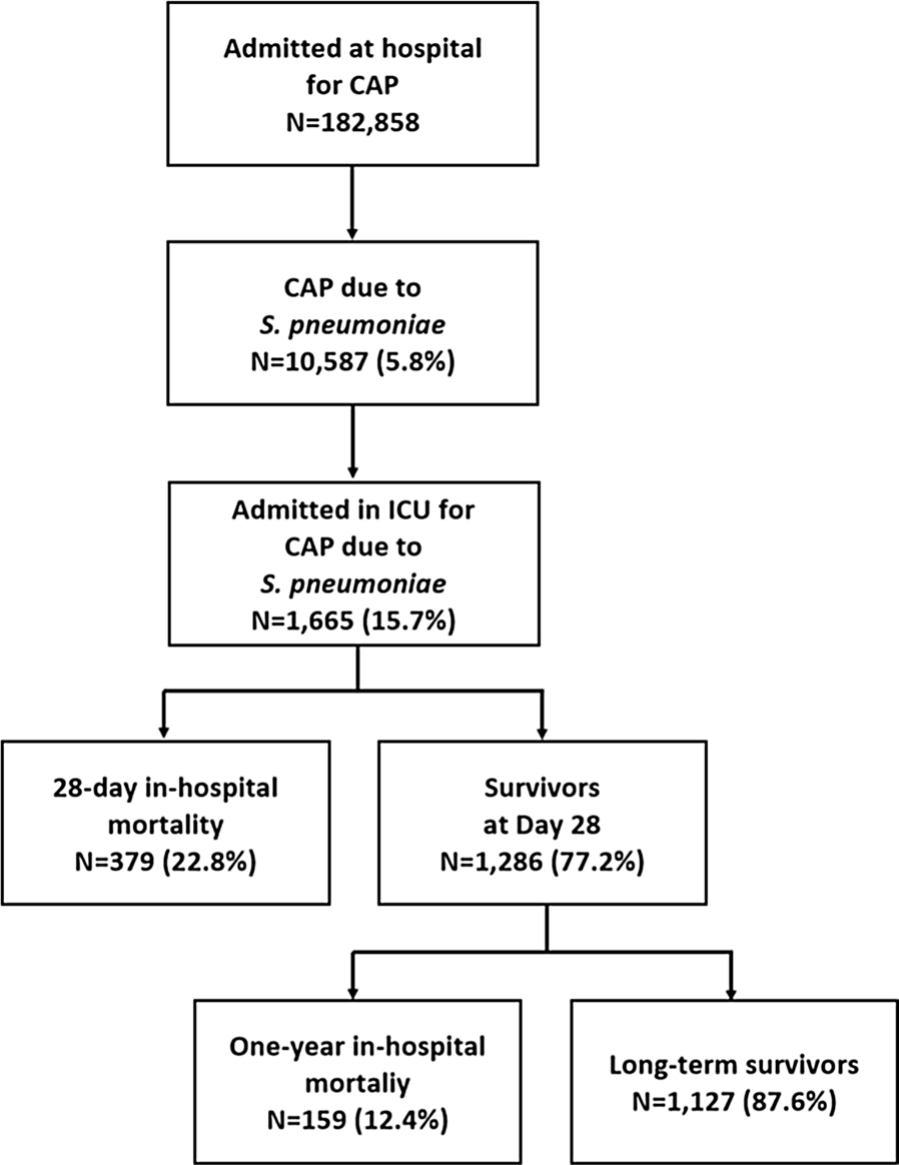
Patient flowchart. CAP: community-acquired pneumonia; *S*. *pneumoniae: Streptococcus pneumoniae*; ICU: intensive care unit

Of a total of 182,858 patients hospitalized for CAP in France during the year 2014 and without any hospitalization for pneumonia within the previous 3 months, 10,587 (5.8%) patients had P-CAP, from which a small minority of patients required ICU admission (1665 patients, 0.9% of all patients with CAP; 15.7% of all patients with P-CAP) (Fig. 1). On the basis of the French population in 2014, the incidence of ICU-admitted patients with P-CAP was 2.53 per 100,000 populations and 5.97 per 100,000 populations over 50 years of age.

Table 1 depicts the main characteristics of ICU-admitted patients with P-CAP. Patients were mostly men (1108/1,665, 67%), of median age 65 years [Q1–Q3 interquartile range (IQR), 55–76]. Only two patients were below 18 years of age. Almost all (1579, 94.8%) had at least one comorbidity; most frequently chronic respiratory diseases (*n* = 861, 51.7%), chronic cardiac failure (*n* = 518, 31.1%), malignant disease (both solid tumors and hematological malignancies) (497, 29.8%) and diabetes (415, 24.9%). Alcohol and/or tobacco abuse was reported in 740 (44.4%) patients. At ICU admission, the mean (SD) SAPS II was 48 [19], 62% of patients required vasopressor agents, and 93.1% required ventilation support: mechanical ventilation for 66.2% (1103/1665) of patients for a median of 7 [IQR, 3–14] days and noninvasive ventilation for 59.3% (987/1665) of patients for a median of 3 [IQR, 1–6] days in median. Their median [IQR] ICU and hospital length of stay (LOS) were 8 [4–16] and 17 [10–29] days, respectively. Among survivors of the initial stay, the median ICU and hospital LOS was 8 [4–15] and 18 [11–30] days, respectively. The median LOS in the ICU was similar across age-groups at 7 [4–16] days below 55 years old, 9 [5–18] days for those aged 55–64 years, 9 [5–16] days for those aged 65–75 years, and 7 [4–14] days for those older than 75 years.

### Outcomes

Patients with an early death (379 patients, 22.8%) were more often men (72% vs. 65% in survivors) and older (median age 71 [60–80] vs. 64 [53–75] years).

Overall, 516 (31.0%) patients were readmitted at hospital within the following year, most of them once (300, 18.0%). However, 25 (1.5%) were readmitted 4 times, and 25 (1.5%) patients were admitted 5 times or more. Most of the readmissions were due to diseases of the respiratory system (73.7%, lower respiratory tract diseases for one-third of them) and circulatory system (13.6%). The mean (SD) cumulated duration of the stays during the following year was 10.7 (14.3) days. Although the mean number of stays was rather similar regardless of their age-group, the cumulative duration increased with age, from 9.0 (10.9) days for the 55-to-64 age-group to 11.3 (13.7) days for the 65-to-75 age-group (further data in Additional file 1: e-Table 1)**.**

**Fig. 2:**
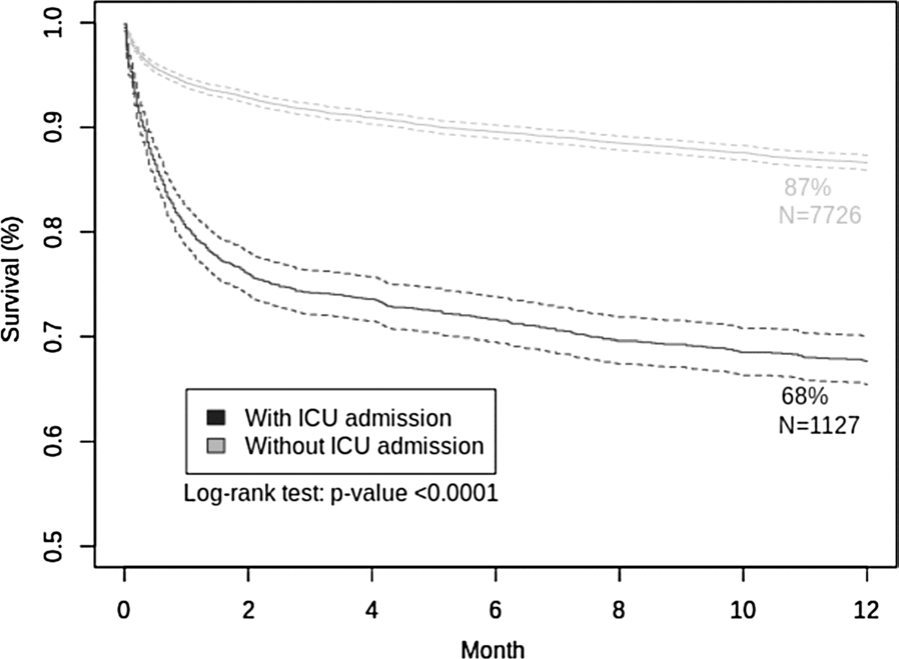
One-year survival curve of patients with pneumococcal community-acquired pneumonia admitted in the intensive care unit (ICU; *n* = 1665) versus those who did not require admission in the ICU (*n* = 8922). Kaplan–Meier estimates of the 1-year mortality: 0.6769 (95% CI 0.6997; 0.6548) for patients admitted in the ICU, versus 0.8619 (95% CI 0.8724; 0.8515) for those not requiring ICU admission (log-rank test *p* value < 0.0001)

Within the following year, the in-hospital mortality rate among ICU-admitted patients was 32.3% (538/1665), compared with 13.4% (1196/8922) of those hospitalized not admitted in ICU, and their in-hospital mortality occurred earlier, at a median [IQR] of 0.7 [0–2] months versus 1.7 [0–5] months for the other inpatients. Among ICU-admitted P-CAP patients, 70.4% of deaths occurred within the first 28 days and 9.5% during the second and third months (e-Fig. 1). The Kaplan–Meier estimate of the 1-year in-hospital survival was 67.7% (95% CI 65.5%–70.0%) for ICU-admitted patients versus 86.2% (95% CI 85.2%–87.2%) for non-ICU-admitted patients (log-rank test *p* < 0.0001) (Fig. 2). The increase in the risk of death of ICU-admitted patients persisted up to 1 year for survivors to the first stay (see Additional file 1: e-Fig. 1).

The illness severity at ICU admission increased the 28-day but not 1-year mortality. In contrast, the factors that independently impacted both short- and long-term mortality were age above 54 years, malignant diseases (HR 1.54, 95% CI [1.23–1.94], *p* = 0.0002, and HR 1.66, 95% CI [1.27–2.18], *p* = 0.0002, respectively) and chronic liver diseases (HR 2.08, 95% CI [1.61–2.69], *p* < 0.0001, and HR 1.80, 95% CI [1.28–2.53], *p* < 0.01, respectively) (Table 2).

### Direct costs associated with the initial and subsequent hospital stays

For all ICU-admitted patients, the mean (SD) cost of the initial hospital stays was €19,008 (€17,218) (Additional file 1, e-Table 2). The mean (SD) cost of the initial stay was similar among those who survived or died at one-year (€18,613 (€16,013) vs. €19,834 (€19,491); Additional file 1, e-Table 2). The mean (SD) cost of the subsequent hospital stays following the initial ICU admission for P-CAP was €11,637 (€16,500) for the subset of patients who were subsequently re-hospitalized.

Compared with that of patients aged 18–54 years, the cost of the initial stay was 11% higher for those aged 55–64 years, whereas it was 11% lower for the patients aged ≥ 76 years. The cost of the subsequent hospital stays was also lower in both higher age-groups compared with that of patients aged 18–54 years: by 28% for patients aged 55–64 years and 14% for those aged ≥ 76. The mean cost per month of life gained increased with age (Additional file 1, e-Table 2).

The 1-year hospital stays among survivors showed that a moderate illness severity at ICU admission was independently associated with increased costs (+ 22% (beta index 0.20, 95% CI [0.11–0.29]), whereas costs were significantly decreased for the most severely ill patients (− 19% (− 0.21, 95% CI [− 0.22: − 0.10], *p* = 0.0003) (Table 3). Chronic cardiac failure and chronic respiratory diseases were independent risk factors for increased costs.

**Table 3:** Determinants of the costs associated with patients with pneumococcal community-acquired pneumonia and admitted in intensive care unit, overall and among the one-year survivors

	All patients (*n* = 1665)		Multivariable model					One-year survivors (*n* = 1127)		Multivariable model				
	*N*	(%)	Beta	95% confidence interval		*p* Value	% of cost increase	*N*	(%)	Beta	95% confidence interval		*p* Value	% of cost increase
Intercept			9.97	9.80-	10.14	< 0.0001								
Age (years)						< *0.01*								
0–54	394	(24)	Ref	–	–	–	–	322	(29)	Ref	–	–	–	–
55–64	409	(25)	− 0.02	− 0.12	0.08	0.7299		290	(26)	0.00	− 0.09	0.10	0.9291	
65–75	424	(25)	0.01	− 0.09	0.11	0.7966		277	(25)	0.00	− 0.11	0.10	0.9265	
> 76	438	(26)	− 0.19	− 0.30	− 0.09	0.0002	− 17	238	(21)	− 0.15	− 0.25	− 0.04	0.0075	− 14
Sex (ref: women)	1108	(67)	0.10	(0.03-	0.17)	0.0066	11	730	(65)	0.07	0.00	0.15	0.0559	7
SAPSII initial stay													< *0.0001*	
0–34	872	(52)	Ref.	–	–	–				Ref	–	–	–	–
35–46	352	(21)	0.20	0.11	0.29	0.0000				0.27	0.18	0.35	< 0.0001	30
47–60	254	(15)	0.07	− 0.03	0.17	0.1593				0.27	0.16	0.38	< 0.0001	31
> 61	187	(11)	− 0.21	− 0.33	− 0.10	0.0003				0.42	0.27	0.57	< 0.0001	52
Comorbidities														
Cardiac failure	518	(31)	0.14	0.06	0.22	0.0004	15	297	(26)	0.10	0.02	0.19	0.0189	11
Chronic respiratory diseases	861	(52)	0.11	0.04	0.18	0.0023	12	594	(53)	0.10	0.03	0.18	0.0062	11

SAPS: Simplified Acute Physiological Score

## Discussion

This study describes a comprehensive survey of all patients with pneumococcal pneumonia hospitalized in the ICU in France for 1 year and the associated burden in terms of in-hospital mortality and direct costs. Although many studies on patients with pneumococcal pneumonia are focused on the risk factors for ICU admission and the 28-day mortality rates, this study broadened the scope to the risk factors for increased mortality and costs during both the initial and all the subsequent hospital stays within the year following the ICU admission. We showed that the early mortality was correlated with the initial illness severity at ICU admission, age, and two comorbidities, malignant diseases and chronic liver diseases, whereas the initial illness severity was no longer a risk factor for 1-year mortality. The in-hospital costs were decreased in patients with early mortality, i.e., the most severely ill at admission. Unlike mortality, the comorbidities that impacted in-hospital costs were chronic cardiac and respiratory diseases.

The 28-day mortality rate of our study is consistent with that reported by others. In a multicenter French study on 614 ICU-admitted patients with severe P-CAP, the mortality rate was 18.9% [11]. Age, gender, and organ failures at ICU admission were more strongly associated with hospital mortality than comorbidities as expressed through the Charlson index [11]. This population was slightly less severely ill, as their median SAPS II at ICU admission was at 43 [IQR 32–57], whereas our patients had a median SAPS II at 48, and more than 60% were mechanically ventilated and required vasopressor agents. The mortality rate of 29% observed in another French study on invasive pneumococcal infections, mainly P-CAP, comprised 48% severely ill patients, of which 31% were admitted in ICUs [12]. Another French study on ICU patients with invasive pneumococcal infection reported a similar 28-day mortality rate of 19.8% [13]. Diabetes mellitus was the only comorbidity independently associated with increased mortality (odds ratio 1.91, 95% CI [1.23–3.03], *p* = 0.006), in that study that contrasts to our study where diabetes had no prognostic value in multivariate analysis. The frequent complication of diabetes with chronic cardiovascular diseases may explain why diabetes itself is not eventually associated with prognosis in our multivariate model.

After the initial ICU stay, the 1-year in-hospital mortality was 12% and increased with age and comorbidities including malignant and liver diseases. Our observed mortality is similar to the overall 1-year post-ICU mortality reported by a Dutch study [14] but lower than that of a US study after ICU stay for sepsis [15] and a Finnish study in ICU patients with severe CAP [16]. The prognostic value for the long-term mortality of any type of preexisting comorbidities was also reported by others [17–22]. More specifically, our study showed that the 1-year mortality is correlated with some comorbidities rather than with the illness severity at ICU admission, as already highlighted by others [23–26]. A French study on the 1-year survival of ICU survivors reported an OR at 1.65 [95% CI 1.13–2.42] for patients with active malignant disease [24]. Another study reported a hazard ratio of 1.98 [95% CI 1.17–3.37] for the long-term mortality in hospitalized patients with CAP with cancer [25]. Similarly, the independent impact of chronic liver diseases on the mortality of ICU patients was already described [23, 26]. However, the impact of these severe complications on the 1-year mortality might be more related to their own severity than to the CAP episode that triggered the initial hospital admission. Of note, cardiac events, frequently reported as jeopardizing the medium- and long-term outcomes of hospitalized CAP patients [17, 21, 27], were not a major cause of readmissions.

In our study, the mean cost of the initial ICU stay was slightly above €19,000. In a previous study conducted in France in 2011–2014 which evaluated the direct costs associated with P-CAP, the cost for the subset of the ICU-admitted patients was €14,385, that is, lower than observed in our study [10]. With regard to age, the costs in our study ranged approximately €18–20,000 for the initial stay and were slightly lower for patients older than 75 years, which may be explained by the higher early mortality in this age-group. Interestingly, the costs of the subsequent stays for the younger adults (18–54 years) were twice as high as those for the oldest adults (> 75 years). Importantly, on its own, the high age was not an independent risk factor for increased costs in our study. Because of the differences in health systems, costs differ from one country to another, and direct comparisons must be made with caution.

The 1-year hospital costs were closely linked to rehospitalizations and were experienced by one patient out of three in our study. This rate is lower than the 40% readmission rate within 3 months observed in the USA for sepsis [16], and the overall 72% readmission rate observed for hospitalized CAP patients [28]. It may reflect the French policy for ICU admission of patients that selects patients who will benefit the most from their ICU stay with a better intermediate-term prognosis. Importantly, chronic cardiac and respiratory diseases were associated with a substantial increase in 1-year costs, and readmissions were by far mostly due to these two comorbidities. Of note, the proportion of readmission due to chronic respiratory diseases was higher than the proportion of this comorbidity in our population, suggesting that the ICU admission for P-CAP may be associated with a worsening of the underlying respiratory diseases. The respective impact of P-CAP and chronic illness by itself on the readmissions was not addressed by our study.

In our study, specific comorbidities but not age were independent prognosis factors for the pneumococcal disease burden, that supports French recommendations, which are solely comorbidities driven for adult patients [29]. This is in contrast to other guidelines for which age directs vaccination recommendations [30, 31]. The four comorbidities that we identified as drivers for an increased CAP burden are included in most immunization recommendations. However, malignant diseases, which are all included in the recent French recommendations [32], are often limited elsewhere to some subpopulations, such as metastatic stage, or some specific hematological malignancies. Patients with cancer, although at higher risk of increased 1-year in-hospital mortality, as observed in our study and others [33], exhibit a low rate of pneumococcal vaccination [34, 35]. A recent French study showed a 10% rate among patients with comorbidities at risk [12]. A single-institution study focusing on 99 patients with gastrointestinal cancer yielded a pneumococcal vaccination rate of 10.1% (95% CI [4.1–16]) [36]. As shown in several studies, childhood vaccination programs contribute to herd immunity, thus partly protecting non-vaccinated adults [37, 38]. However, this protection of adults has been deemed insufficient in France, despite a high coverage of vaccination among children [39]. Vaccination among adults including those older than 65 years has been shown to be efficient [40]. Healthcare professionals, cancer societies, and other societies advocate for increasing this vaccination rate, which some vaccination programs increased successfully [36]. Cardiovascular diseases are the leading cause of mortality of diabetic patients [41], which may explain the lack of a signal due to diabetes mellitus in our model. Beyond specific comorbidities, the immunization coverage reported for elderly residents from French long-term nursing facilities was low at 27% (95% CI [21–34]) among those with targeted comorbidities and 17% (95% CI [14–20]) overall [42].

Despite its strengths, this study has some limitations. It relies on an administrative database that mainly has budgetary purposes. Therefore, the coding of some diseases such as the P-CAP diagnosis might be suboptimal. The reliability is not that of a clinical database monitored against source data. However, we used ICD-10 of the coding system, which showed more reliability for the pneumonia diagnosis, and we combined codes for an improved accuracy [43, 44]. In addition, some types of data were not available, such as the intensity of signs and symptoms at admission; the use of some resources, such as the dialysis procedures; or the immunization status of the patients. However, as discussed earlier, the rate of vaccinated people is low in France, even among at-risk patients. It is another limitation that we did not have the tests used and their results and microbiological data related to the susceptibility profile of the serotype of the pneumococcal strains, as well as the adequacy of antimicrobial therapy.

Another limitation is that this database collects solely in-hospital data. Therefore, we do not have any insight regarding what happened outside of the hospital, such as the out-of-hospital mortality rate. However, a French study showed that the rate of in-hospital deaths in 2008 ranged approximately 60% of deaths for the patients aged between 40 and 89 years and that these rates were stable over 15 years [45]. Therefore, we can evaluate our rate of missing death-related data at approximately 40% of our known decedents. In addition, solely data on acute hospital stays were collected, and we did not collect whether the patient was discharged home or to a rehabilitation facility. Another limitation is the lack of indirect cost data, e.g., those related to ambulatory consultations, drugs, and medical procedures. Finally, while potentially several control groups would have been of interest, such as patients admitted in the ICU with CAP not due to *S. pneumoniae*, or pneumococcal invasive infections other than CAP, or other reasons besides CAP patients for being transferred into the ICU.

## Conclusions

Although severe pneumococcal pneumonia in France was associated with a survival of 81% at discharge, the Kaplan–Meier estimates of the 1-year survival were 68%. The 1-year prognosis was mainly influenced by age and comorbidities, such as chronic liver diseases and malignant diseases. The burden of severe pneumococcal pneumonia is not only a heavy mortality but also a high rate of new hospital stays within the year following the pneumonia onset. Overall, the in-hospital costs within the year following pneumonia were higher than €22,000 and significantly increased in patients with cardiac diseases and chronic respiratory diseases. Overall, severe pneumococcal pneumonia in patients with chronic comorbidities, such as malignant diseases, chronic liver, and cardiac and respiratory diseases, is associated with a heavy 1-year burden, and it might be prevented by a better compliance to the current guidelines for pneumococcal vaccination.

## Supplementary information

**Additional file 1.** Further details on Methods, on data regarding the hospital stays (e-Table 1 and e-Table 2), and on the 1-year survival curves of patients (e-Fig. 1). In Methods, further details are provided on the codes used for the identification of the pneumonia diagnosis, of the comorbidities and procedures used, and of the pneumonia etiology. **e-Table 1**. Characteristics of the new hospital stays within the year following the CAP-related hospitalization, overall and according to the age group, among those with at least one new hospital stay. **e-Table 2**. Direct costs of the initial hospital stay and of the subsequent hospital stays within the first year, by patient with pneumococcal communityacquired pneumonia and admitted in intensive care unit, overall, according to the one-year outcome, and by age group. **e-Fig. 1**. Comparative survival curves of patients with pneumococcal pneumonia (P-CAP) admitted in intensive care unit (ICU), (A) from the hospitalization to 3 months after (3-month survivors: 74.2% (1,235/1,665) ICU-admitted patients vs. 91.7% (8,179/8,922) non ICU-admitted patients); (B), among the 3-months survivors, from 3 to 12 months after the hospitalization (91.3% (1,127/1,235) ICU-admitted patients vs. 94.5% (7,726/8,179) non-ICU-admitted patients).

## Data Availability

The datasets used and/or analyzed during the current study are available from Pfizer's authors on reasonable request.
