# Supplementary material for: Burden of pneumococcal pneumonia requiring ICU admission in France: 1-year prognosis, resources use, and costs
Source: Crit Care. 2021 Jan 10;25:24. doi: 10.1186/s13054-020-03442-z (PMC7798246; doi:10.1186/s13054-020-03442-z)

Additional File 1

Burden of Pneumococcal pneumonia requiring ICU admission in France: 1-year prognosis, resources use and costs

Claire Dupuis[1,2,3,*], Ayman Sabra[4,*], Juliette Patrier[1], Gwendoline Chaize[5], Amine Saighi[4], Céline Féger[6], Alexandre Vainchtock[5], Jacques Gaillat[7], Jean-François Timsit[1,3]

Methods

Identification of diagnosis, comorbidities and procedures

The codes from the International Statistical Classification of Diseases, 10th Revision (ICD-10) which were used for the principal diagnosis of CAP are detailed below, as are those which were used for the comorbidities**.** Comorbidities were collected using the hospital stays within the 5 previous years. Associated diagnoses might be added in case of concomitant disease that increases the burden of care, of if it was managed during the hospital stay in addition of the management of the main diagnosis. All the procedures were based on the CCAM codes (French acronyms for *Classification Commune des Actes Médicaux*, i.e., General Coding of Medical Acts).

Codes used for pneumonia diagnosis

Detailed codes used for pneumonia diagnosis: A212 Pulmonary tularemia; A221 Lung anthrax; A370 ; Whooping cough caused by Bordetella pertussis; A371 Whooping cough caused by Bordetella parapertussis; A378 Whooping cough caused by other Bordetella species; A379 Whooping cough, unspecified; A420 Pulmonary actinomycosis; A430 Pulmonary nocardiosis ; A481 Legionnaires' disease (pulmonary); A70 Chlamydia psittaci infection; A78 Q fever; B012 Varicella pneumonia (J17.1 *); B052 Measles complicated by pneumonia (J17.1 *); B068 Rubella with other complications; B250 Cytomegalovirus pneumonia (J17.1 *); B371 Pulmonary candidiasis; B380 Acute pulmonary coccidioidomycosis; B382 Pulmonary coccidioidomycosis, unspecified; B390 Acute pulmonary histoplasmosis caused by Histoplasma capsulatum; B391 Chronic pulmonary histoplasmosis caused by Histoplasma capsulatum; B392 Pulmonary histoplasmosis caused by Histoplasma capsulatum, unspecified; B393 Histoplasmosis, disseminated caused by Histoplasma capsulatum; B394 Histoplasmosis caused by Histoplasma capsulatum, unspecified; B395 Histoplasmosis caused by Histoplasma duboisii; B399 Histoplasmosis, unspecified; B440 Invasive pulmonary aspergillosis; B441 Other pulmonary aspergillosis; B583 Pulmonary toxoplasmosis (J17.3 *); B59 Pneumocystosis; J100 Influenza with pneumonia, other influenza virus identified; J110 Influenza with pneumonia, unidentified virus; J120 Adenoviral pneumonia; J121 Respiratory syncytial virus [RSV] lung disease; J122 Pneumopathy caused by para-influenza virus; J123 Pneumonia caused by human metapneumovirus; J128 Other viral pneumonia; J129 Viral pneumonia, unspecified; D13 Pneumonia caused by Streptococcus pneumoniae; J14 Pulmonary disease caused by Haemophilus influenzae; J150 Pneumonia caused by Klebsiella pneumoniae; J151 Pneumonia caused by Pseudomonas; J152 Pneumonia caused by staphylococci; J153 Pneumonia caused by streptococci, group B; J154 Pneumonia caused by other streptococci; J155 Pneumonia caused by Escherichia coli; J156 Pneumonia caused by other aerobic Gram-negative bacteria; J157 Pneumonia caused by Mycoplasma pneumoniae; J158 Other bacterial pneumonia; J159 Bacterial pneumonia, unspecified; J160 Pneumonia caused by Chlamydia; J168 Pneumonia caused by other infectious microorganisms; J170 Pulmonary disease in bacterial diseases classified elsewhere; J171 Pulmonary disease in viral diseases

classified elsewhere; J172 Pneumopathy during mycosis; J178 Pneumonia in other diseases classified elsewhere; J180 Bronchopneumopathy, unspecified; J181 Lobar pulmonary disease, unspecified; J188 Other pneumonia, unspecified microorganism; J189 Pneumopathy, unspecified; J690 Pneumonia due to food and vomiting; J850 Gangrene and lung necrosis if specified infectious; J851 Lung abscess with pneumonia.

Codes used for diagnosis of pneumococcal etiology: J13 Pneumonia caused by *Streptococcus pneumoniae*, and B953 Other diseases classified elsewhere caused by *Streptococcus pneumoniae*.

Codes used for secondary diagnosis of pneumonia: B206, B24+9, C340, C341, C342, C343, C349, C780, D022, D143, D381, I200, I200+0, I201, I209, I210, I2100, I21000, I2108, I211, I2110, I21100, I2118, I212, I2120, I21200, I2128, I2130, I21300, I2138, I2140, I21400, I2148, I219, I2190, I21900, I2198, I2200, I2208, I2210, I22800, I500, I501, I509, J09, J101, J108, J111, J118, J173, J182, J440, J441, J448, J449, J450, J451, J458, J459, J46, J679, J691, J698, J704, J80, J81, J840, J841, J849, J860, J869, J90, J91, J930, J931, J938, J939, J960*, J961*, J969, R042, R060, R071, R072, R074, R91, Z512.

Codes used for the comorbidities

Alcohol abuse: F10, and tobacco abuse: F17.

High-risk comorbidities:

HIV: B20 to B24, Z21; asplenia/hyposplenia: D56, D57, D73; immune disorders and auto-immune diseases: D80 to D84, D89, K50, K51, L40, L93, M05 to M09, M32, M45 to M49.

Solid tumors: C00 to C26, C30 to C34, C37 to C41, C43 to C58, C60 to C80, C97, D00 to D07, D09, D37 to D44, D48; malignant hemopathies: C81 to C86, C88, C90 to C96, D46, D47, D61.

Transplant: T86, Z94, Z95

At-risk comorbidities:

Diabetes mellitus: E10 to E14

Cardiac diseases: I05 to I11, I13, I15, I20 to II25, I27, I30 to I32, I34 to I37, I40 to I49, I51, I52; cardiac insufficiency: I50.

Respiratory diseases: J41 to J43, J47, J960 (if asthma is associated); including chronic obstructive bronchial disease: J44, asthma: J45 and J46, and respiratory insufficiency J961

Liver diseases: K73, K75, K76; including liver failure: K72, and cirrhosis: K70 and K74.

Renal diseases: I12, N03, N04, N08, N11, N14, to N16, N19; including chronic renal failure: N18, Z49, Z992

Data collected

The available collected data on the hospital stay were the length of stay in days and the month of discharge, whether the patient was admitted in ICU, and the length of the ICU stay. Comorbidities were also coded.

For each initial hospital stay, the following individual de-identified data were obtained: demographics (age, gender), hospital characteristics (teaching versus non-teaching and private versus public hospital), patient’s comorbidities according to ICD-10, and the severity of the patients on ICU admission (organ failures, and a general severity score such as SAPS II). Regarding the ICU stay, the number of days with each type of organ support such as mechanical ventilation, renal replacement therapy and inotropes are recorded. The length of the initial hospital stay and in-hospital mortality were also recorded.

For each patient, the characteristics of the subsequent hospital stays within the year following the initial stay were also collected, as were the alcohol and tobacco consumption reported by the patient.

Costs evaluation: variables collected

Items of healthcare consumption taken into account are hospitalizations and medications. Standard medication and device costs are included in the GHM (French acronym for "*Homogeneous Group Stays"*) unit cost for hospitalization. Expensive in-hospital drugs (from "*Liste en sus*", i.e., additional list) are reported in a specific database (FICHCOMP database) for each hospital stay. These sources are integrated into our assessment of hospitalization cost. Medications and devices prescribed or delivered during outpatient visits are not be considered.

Costing are restricted to direct costs and determined from the perspective of the French social security system. Costs are attributed from official French national tariffs from 2014 to 2015, and expressed in 2016 Euros. A standard national tariff is applied to each hospitalization based on the GHM code attributed in the PMSI database. GHM tariffs include medical and related procedures, nursing care, treatments (except specific expensive drugs and implants), drugs/devices used, food and accommodation, and investment costs for hospitalized patients. The additional cost per day of hospitalization in an intensive care unit are added to GHM tariffs, when appropriate. Expensive drugs are costed using the public retail price issued from the FICHCOMP database. For private hospitals, physician's fees are also added to the GHM tariffs as physicians are paid on a fee-for-service basis (source: ENCC, French acronym for French Reference for Costs of Common Methodology, or *"Echelle Nationale des Coûts à méthodologie Commune"*, a tool that links the medical administrative data from the PMSI and the financial data from hospitals). For public hospitals, physicians being salaried, their wages are included inside the GHM tariff.

e-Table 1 - Characteristics of the new hospital stays within the year following the CAP-related hospitalization, overall and according to the age group, among those with at least one new hospital stay

	Overall population	Older patients		All age groups				
		> 55 y.o.	> 76 y.o.	0-17 y.o.	18-54 y.o.	55-64 y.o.	65-75 y.o.	≥ 76 y.o.
**No. of patients**	513	397	125	1	103	125	149	135
Number of new hospital stays								
Mean (SD)	1.9 (1.5)	1.8 (1.5)	1.7 (1.1)	4.0 (NA)	2.0 (1.6)	1.9 (2.0)	1.9 (1.3)	1.7 (1.1)
Median	1	1	1	4	1	1	1	1
Maximum	13	13	6	4	10	13	7	6
Cumulated duration of the new hospital stays (days)								
Duration (days)	962	735	216	4	209	239	285	225
Mean (SD)	10.7 (14.3)	10.5 (12.1)	11.0 (10.8)	14.5 (6.1)	11.3 (20.5)	9.0 (10.9)	11.3 (13.7)	10.9 (10.9)
Median	7	7	8	16	7	6	8	8
Maximum	240	112	92	20	240	68	112	92

e-Table 2 – Direct costs of the initial hospital stay and of the subsequent hospital stays within the first year, by patient with pneumococcal community-acquired pneumonia and admitted in intensive care unit, overall, according to the one-year outcome, and by age group.
The costs of the initial stay are calculated for the full study population (n=1;665 patients). The costs of the subsequent hospital stays within the following year are calculated among the patients alive at ICU discharge of their initial stay and who required to get hospitalized during that year following their P-CAP episode (n=513).

	Number	Mean	SD	Median	Q1	Q3
Costs of the initial hospital stay						
**All patients**	1,665	19,008 €	17,218 €	14,244 €	9,900 €	22,752 €
**All patients, by outcome**						
**1-year survivors**	1,127	18,613 €	16,013 €	14,244 €	9,938 €	22,038 €
**1-year decedents**	538	19,834 €	19,491 €	14,279 €	9,348 €	24,562 €
**All patients, by age group**						
**18-54 years**	394	18,556 €	15,365 €	13,994 €	9,537 €	22,821 €
**55-64 years**	409	20,216 €	19,163 €	15,528 €	10,214 €	23,748 €
**65-75 years**	424	20,065 €	16,950 €	15,030 €	10,149 €	23,351 €
**≥ 76 years**	438	17,263 €	17,025 €	12,406 €	9,411 €	20,016 €
Costs of the subsequent stays						
**All patients**	513	11,637 €	16,500 €	6,658 €	3,485 €	13,702 €
**All patients, by outcome**						
**1-year survivors**	538	19,834 €	19,491 €	14,279 €	9,348 €	24,562 €
**1-year decedents**	113	14,815 €	23,743 €	7,605 €	4,284 €	18,244 €
**All patients, by age group**						
**18-54 years**	104	16,171 €	26,614 €	7,115 €	3,344 €	19,632 €
**55-64 years**	125	10,287 €	11,947 €	5,127 €	2,806 €	14,424 €
**65-75 years**	149	12,001 €	15,345 €	7,004 €	3,543 €	13,683 €
**≥ 76 years**	135	8,991 €	8,873 €	6,106 €	3,800 €	10,327 €
**Cost per month of life gained (initial and subsequent stays)**						
**18-54 years**	394	11,152 €	38,273 €	1,724 €	988 €	3,385 €
**55-64 years**	409	12,425 €	32,037 €	1,954 €	1,259 €	6,784 €
**65-75 years**	424	13,393 €	20,027 €	2,414 €	1,253 €	19,872 €
**≥ 76 years**	438	16,241 €	22,248 €	2,479 €	1,191 €	31,462 €

e-Fig. 1 – Comparative survival curves of patients with pneumococcal pneumonia (P-CAP) admitted in intensive care unit (ICU), (A) from the hospitalization to 3 months after (3-month survivors: 74.2% (1,235/1,665) ICU-admitted patients vs. 91.7% (8,179/8,922) non ICU-admitted patients); (B), among the 3-months survivors, from 3 to 12 months after the hospitalization (91.3% (1,127/1,235) ICU-admitted patients vs. 94.5% (7,726/8,179) non-ICU-admitted patients).

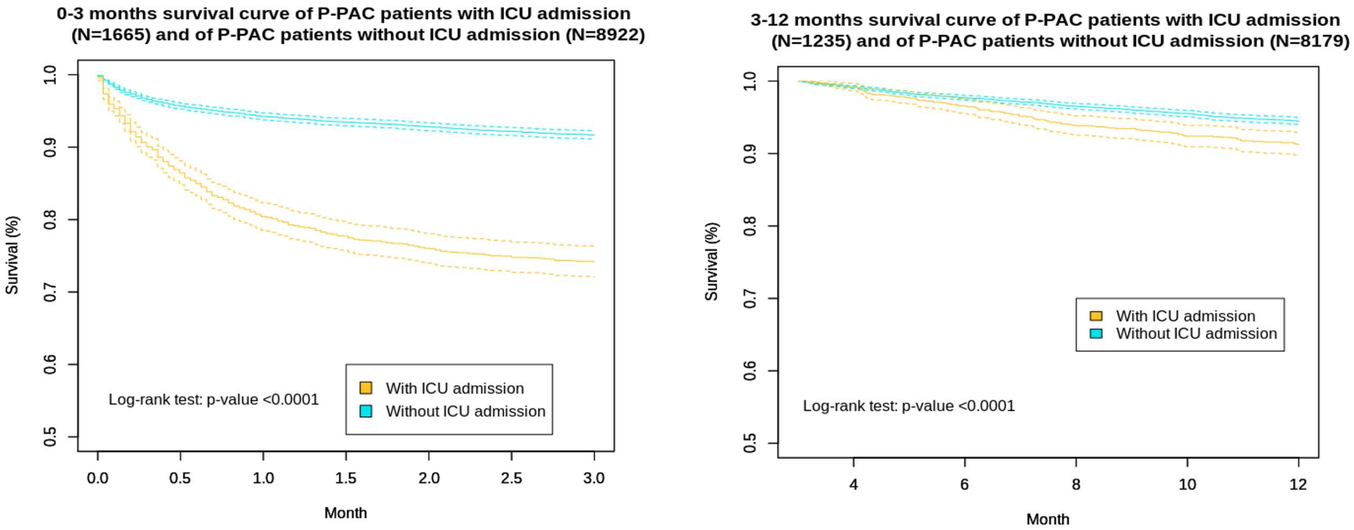

Supplement: Supplementary file 1 — Additional file 1. Further details on Methods, on data regarding the hospital stays (e-Table 1 and e-Table 2), and on the 1-year survival curves of patients (e-Fig. 1). In Methods, further details are provided on the codes used for the identification of the pneumonia diagnosis, of the comorbidities and procedures used, and of the pneumonia etiology. e-Table 1. Characteristics of the new hospital stays within the year following the CAP-related hospitalization, overall and according to the age group, among those with at least one new hospital stay. e-Table 2. Direct costs of the initial hospital stay and of the subsequent hospital stays within the first year, by patient with pneumococcal communityacquired pneumonia and admitted in intensive care unit, overall, according to the one-year outcome, and by age group. e-Fig. 1. Comparative survival curves of patients with pneumococcal pneumonia (P-CAP) admitted in intensive care unit (ICU), (A) from the hospitalization to 3 months after (3-month survivors: 74.2% (1,235/1,665) ICU-admitted patients vs. 91.7% (8,179/8,922) non ICU-admitted patients); (B), among the 3-months survivors, from 3 to 12 months after the hospitalization (91.3% (1,127/1,235) ICU-admitted patients vs. 94.5% (7,726/8,179) non-ICU-admitted patients). [file 13054_2020_3442_MOESM1_ESM.pdf]
